# Does morbid obesity influence perioperative outcomes after video-assisted thoracic surgery (VATS) lobectomy for non-small cell lung cancer? Analysis of the Italian VATS group registry

**DOI:** 10.1007/s00464-021-08680-y

**Published:** 2021-08-16

**Authors:** Francesco Guerrera, Paraskevas Lyberis, Paolo Olivo Lausi, Riccardo Carlo Cristofori, Roberto Giobbe, Massimo Molinatti, Pier Luigi Filosso, Carlo Curcio, Roberto Crisci, Enrico Ruffini, on the behalf of the Italian VATS Group, Mancuso Maurizio, Pernazza Fausto, Refai Majed, Stella Franco, Argnani Desideria, Marulli Giuseppe, De Palma Angela, Bortolotti Luigi, Rizzardi Giovanna, Solli Piergiorgio, Dolci Giampiero, Perkmann Reinhold, Zaraca Francesco, Benvenuti Mauro Roberto, Gavezzoli Diego, Cherchi Roberto, Ferrari Paolo Albino, Mucilli Felice, Camplese Pierpaolo, Melloni Giulio, Mazza Federico, Cavallesco Giorgio, Maniscalco Pio, Voltolini Luca, Gonfiotti Alessandro, Sollitto Francesco, Ardò Nicoletta Pia, Pariscenti Gian Luca, Risso Carlo, Surrente Corrado, Lopez Camillo, Droghetti Andrea, Giovanardi Michele, Breda Cristiano, Lo Giudice Fabio, Alloisio Marco, Bottoni Edoardo, Spaggiari Lorenzo, Gasparri Roberto, Torre Massimo, Rinaldo Alessandro, Nosotti Mario, Tosi Davide, Negri Giampeiro, Bandiera Alessandro, Baisi Alessandro, Raveglia Federico, Stefani Alessandro, Natali Pamela, Scarci Marco, Pirondini Emanuele, Curcio Carlo, Amore Dario, Rena Ottavio, Nicotra Samuele, Dell’ Amore Andrea, Bertani Alessandro, Tancredi Giorgia, Ampollini Luca, Carbognani Paolo, Puma Francesco, Vinci Damiano, Cardillo Giuseppe, Carleo Francesco, Margaritora Stefano, Meacci Elisa, Paladini Piero, Ghisalberti Marco, Crisci Roberto, Divisi Duilio, Fontana Diego, Della Beffa Vittorio, Morelli Angelo, Londero Francesco, Imperatori Andrea, Rotolo Nicola, Alberto, Viti Andrea, Infante Maurizio, Benato Cristiano

**Affiliations:** 1 Department of Surgical Science, University of Torino, Torino, Italy; 2 Department of Thoracic Surgery, Azienda Ospedaliera Universitaria Città della Salute e della Scienza di Torino, Torino, Italy; 3 Department of Thoracic Surgery, Monaldi Hospital, Napoli, Italy; 4 Department of Thoracic Surgery, University of L’Aquila, L’Aquila, Italy; 5 Thoracic Surgery Unit, “G. Mazzini” Hospital, Teramo, Italy; 6 Corso Dogliotti, 14, 10126 Torino, Italy

**Keywords:** Obesity, Lobectomy, Video-assisted thoracic surgery, Morbidity, Mortality, Lung Cancer

## Abstract

**Objectives:**

Obesity in Europe, and worldwide, has been an increasing epidemic during the past decades. Moreover, obesity has important implications regarding technical issues and the risks associated with surgical interventions. Nevertheless, there is a lack of evidence assessing the influence of obesity on video-assisted thoracic surgery (VATS) lobectomy results. Our study aimed to assess the impact of morbid obesity on perioperative clinical and oncological outcomes after VATS lobectomy using a prospectively maintained nationwide registry.

**Methods:**

The Italian VATS lobectomy Registry was used to collect all consecutive cases from 55 Institutions. Explored outcome parameters were conversion to thoracotomy rates, complication rates, intra-operative blood loss, surgical time, hospital postoperative length of stay, chest tube duration, number of harvested lymph-node, and surgical margin positivity.

**Results:**

From 2016 to 2019, a total of 4412 patients were collected. 74 patients present morbid obesity (1.7%). Multivariable-adjusted analysis showed that morbid obesity was associated with a higher rate of complications (32.8% vs 20.3%), but it was not associated with a higher rate of conversion, and surgical margin positivity rates. Moreover, morbid obesity patients benefit from an equivalent surgical time, lymph-node retrieval, intraoperative blood loss, hospital postoperative length of stay, and chest tube duration than non-morbid obese patients. The most frequent postoperative complications in morbidly obese patients were pulmonary-related (35%).

**Conclusion:**

Our results showed that VATS lobectomy could be safely and satisfactorily conducted even in morbidly obese patients, without an increase in conversion rate, blood loss, surgical time, hospital postoperative length of stay, and chest tube duration. Moreover, short-term oncological outcomes were preserved.

Obesity in Europe, and worldwide, has been an increasing epidemic during the past decades [1, 2]. In particular, in Italy, the obesity rate augments from 6% in 1991 to 10% in 2018 [3].

Increasing obesity incidence represents a noteworthy health problem, particularly considering that it is associated with the risk of developing chronic diseases such as cardiovascular disease, musculoskeletal disorders, type 2 diabetes, hypertension, coronary heart disease, sleep apnea, and kidney failure [2, 4, 5] in addition to a generally decreased life expectancy, principally in the young adult population [6–8]. Moreover, all these conditions could increase the risk of postoperative surgical complications, with mutual boost effects [9, 10]. Additionally, obesity itself has important implications regarding technical issues during the surgical procedure [11, 12].

In the literature, there is emerging evidence on relationship between higher BMI and lung cancer [13]. Indeed, the ratio of overweight and obese patients with lung cancer who present for major pulmonary resection for NSCLC incessantly raised during the last decades [14].

In order to increase perioperative outcomes and decrease postoperative complications, minimally invasive thoracic surgical procedures [i.e., video-assisted thoracic surgery (VATS) lobectomy] have been largely adopted worldwide, in alternative to classic open surgery via thoracotomy [14]. Recently, the results of a UK multicentric VIOLET randomized controlled trial showed a relationship between VATS lobectomy and enhanced postoperative clinical outcome [15]. However, there is a lack of evidence assessing the influence of morbid obesity on VATS lobectomy results.

The aim of our study was to assess the impact of morbid obesity (Body Mass Index ≥ 40) on perioperative clinical and oncological outcomes after VATS lobectomy using a prospectively maintained nationwide registry, the Italian VATS group registry [16].

## Methods

From 2016 to 2019, out of a total of 4972 patients submitted to thoracoscopic major lung resection by 55 centers of the Italian VATS Group database, 4412 patients submitted to VATS lobectomy for lung cancer were included in the present study. Patients submitted to VATS segmentectomy, bilobectomy, or pneumonectomy were excluded. Informed consent was obtained from all individual participants included in the study.

Primary outcomes explored were conversion to thoracotomy rates and complication rates (Grade ≥ 2 according to the Clavien–Dindo classification [17]). An additional analysis on primary endpoints was performed stratifying patients according to BMI as follows: ≤ 30, 30–40, ≥ 40.

As secondary outcomes, surgical margin positivity rates, intra-operative blood loss, surgical time, hospital postoperative length of stay, chest tube duration, and the number of harvested lymph-node were assessed.

### Statistical analysis

Baseline patient characteristics are summarized by number and percentages, or median and interquartile range (IQR), as appropriate. Between-group differences were evaluated by Wilcoxon–Mann–Whitney test (continuous variables) or χ^2^ test or Fisher’s exact test (categorical variables), as appropriate.

Univariable and multivariable-adjusted logistic regression models were used to evaluate categorical outcomes, while different continuous outcomes were compared by the Wilcoxon–Mann–Whitney test. The variables in the adjusted models were age, gender, smoking history, CCI, ECOG performance status, FEV1%, DLCO%, surgeon experience, pT stage, pN stage, preoperative diagnosis, and performed adhesiolysis.

All statistical tests were two-sided and *P* values of 0.05 or less were considered statistically significant. Data analysis was performed using Stata software version 15.1 (Stata-Corp, College Station, Texas).

## Results

Median BMI in the present cohort was 25.8 (IQR 23.4–28.7) (Table 1). Most of the patients present a BMI < 40 (4338–98.3%), while out of 74 patients were morbidly obese (BMI ≥ 40). Most patients were male (2681–60.7%) and the mean age at the time of surgery was 69 years (IQR 62–75). 425 (9.6%) cases of conversion to thoracotomy were observed in the whole population. Median surgical time was 174 min (IQR 135–210), the median number of harvested lymph-nodes was 11 (IQR 7–16), median intraoperative blood loss 100 ml (IQR 50–200), median chest drain duration 4 days (IQR 3–5), and median postoperative length of stay 5 days (IQR 4–7). In the whole cohort, 906 (20.5%) complications and 100 (2.3%) surgical margin positivity cases were observed (Table 2).

**Table 1:** Baseline characteristics in the overall population

Factor	All
	*n* = 4412
BMI, *n* (%)	
Median (IQR^a^)	25.8 (23.4–28.7)
≥ 40	74 (1.7)
< 40	4338 (98.3)
Age (years), median (IQR)	69 (62–75)
Gender (male), *n* (%)	2681 (60.7)
Smoking history (ever), *n* (%)	3137 (71.1)
CCI, median (IQR)	3 (3–4)
ECOG, median (IQR)	0 (0–1)
FEV1 (%), median (IQR)	93 (80–106)
DLCO (%), median (IQR)	83 (71–95)
Surgeon experience^b^*, n* (%)	2676 (60.7)
cT stage, *n* (%)	
cT1a–b–c	2939 (66.6)
cT2a–b	1067 (24.2)
cT3	322 (7.3)
cT4	83 (1.8)
cN stage, *n* (%)	
cN0	3873 (87.9)
cN1	274 (6.3)
cN2	256 (5.8)
pT stage, *n* (%)	
pT1a–b–c	2893 (66.1)
pT2a–b	1054 (24.1)
pT3	336 (7.6)
pT4	97 (2.2)
pN stage, *n* (%)	
pN0	3873 (81.8)
pN1	418 (9.9)
pN2	351 (8.3)
Preoperative diagnosis (yes), *n* (%)	1900 (43.1)
Adhesiolysis (yes), *n* (%)	1218 (27.6)

*BMI* Body Mass Index, *CCI* Charlson comorbidity index, *ECOG* Eastern Cooperative Oncology Group performance status

^a^Interquartile range

^b^ > 50 VATS lobectomy procedures performed

**Table 2:** Baseline characteristics: morbid obesity (BMI ≥ 40) vs Non-morbid obesity (BMI < 40) groups

Factor	BMI ≥ 40	BMI < 40	*P*
	*n* = 74	*n* = 4338	
Age (years), median (IQR^a^)	68 (59–63)	69 (63–75)	0.089
Gender (male), *n* (%)	40 (54.1)	2641 (60.9)	0.233
Smoking history (ever), *n* (%)	50 (67.6)	3087 (71.2)	0.499
CCI, median (IQR)	3 (2–4)	3 (3–4)	0.041
ECOG, median (IQR)	0 (0–1)	0 (0–1)	0.835
FEV1 (%), median (IQR)	93 (82–109)	93 (80–106)	0.884
DLCO (%), median (IQR)	89 (78–100)	83 (71–95)	0.008
Surgeon experience^b^, *n* (%)	39 (52)	2637 (60.8)	0.099
cT stage, *n* (%)			0.103
cT1a–b–c	58 (78.3)	2881 (66.4)	
cT2a–b	14 (18.9)	1053 (24.3)	
cT3	1 (1.4)	321 (7.4)	
cT4	1 (1.4)	82 (1.9)	
cN stage, *n* (%)			0.683
cN0	63 (85.1)	3810 (87.9)	
cN1	7 (9.5)	267 (6.2)	
cN2	4 (5.4)	343 (5.9)	
pT stage, *n* (%)			0.063
pT1a–b–c	57 (77.0)	2836 (65.9)	
pT2a–b	14 (18.9)	1040 (24.1)	
pT3	1 (1.4)	335 (7.8)	
pT4	2 (2.7)	95 (2.2)	
pN stage, *n* (%)			0.584
pN0	55 (77.4)	3406 (81.9)	
pN1	8 (11.3)	410	
pN2	8 (11.3)	343 (8.2)	
Preoperative diagnosis (yes), *n* (%)	2469 (56.9)	43 (58.1)	0.837
Adhesiolysis (yes), *n* (%)	3139 (72.4)	55 (74.3)	0.708

*BMI* Body Mass Index, *CCI* Charlson comorbidity index, *ECOG* Eastern Cooperative Oncology Group performance status

^a^Interquartile range

^b^ > 50 VATS lobectomy procedures performed

**Fig. 1:**
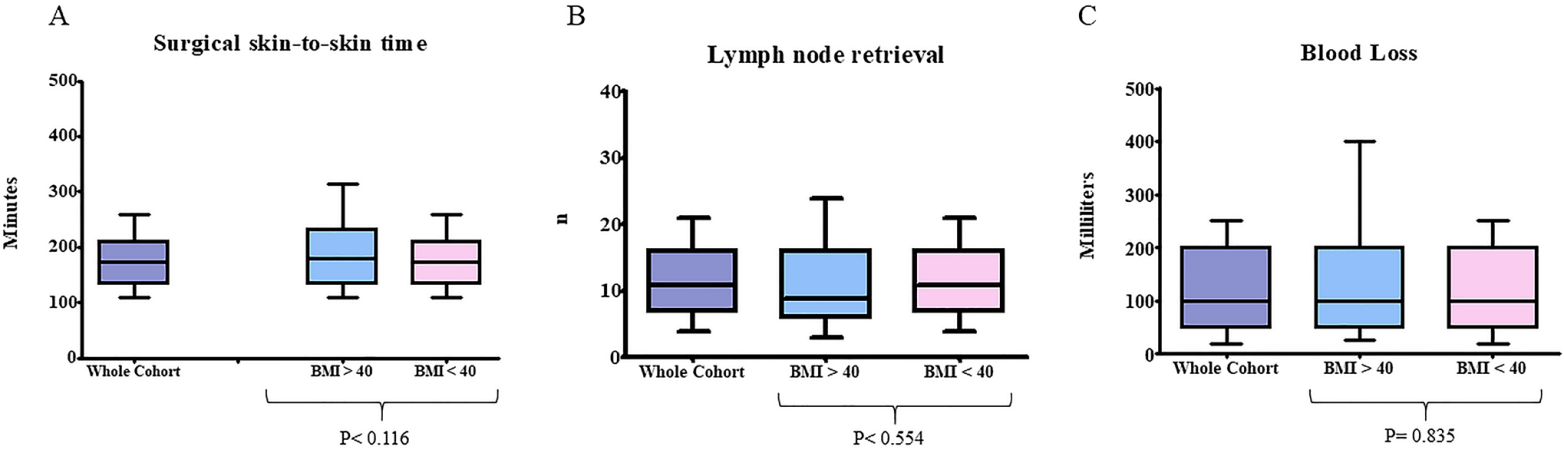
Morbid obesity (BMI ≥ 40) Versus Non-morbid obesity (BMI < 40): Box-and-whisker plots illustrate the distribution of surgical time (**A**), lymph-node retrieval (**B**), and blood loss (**C**)

**Fig. 2:**
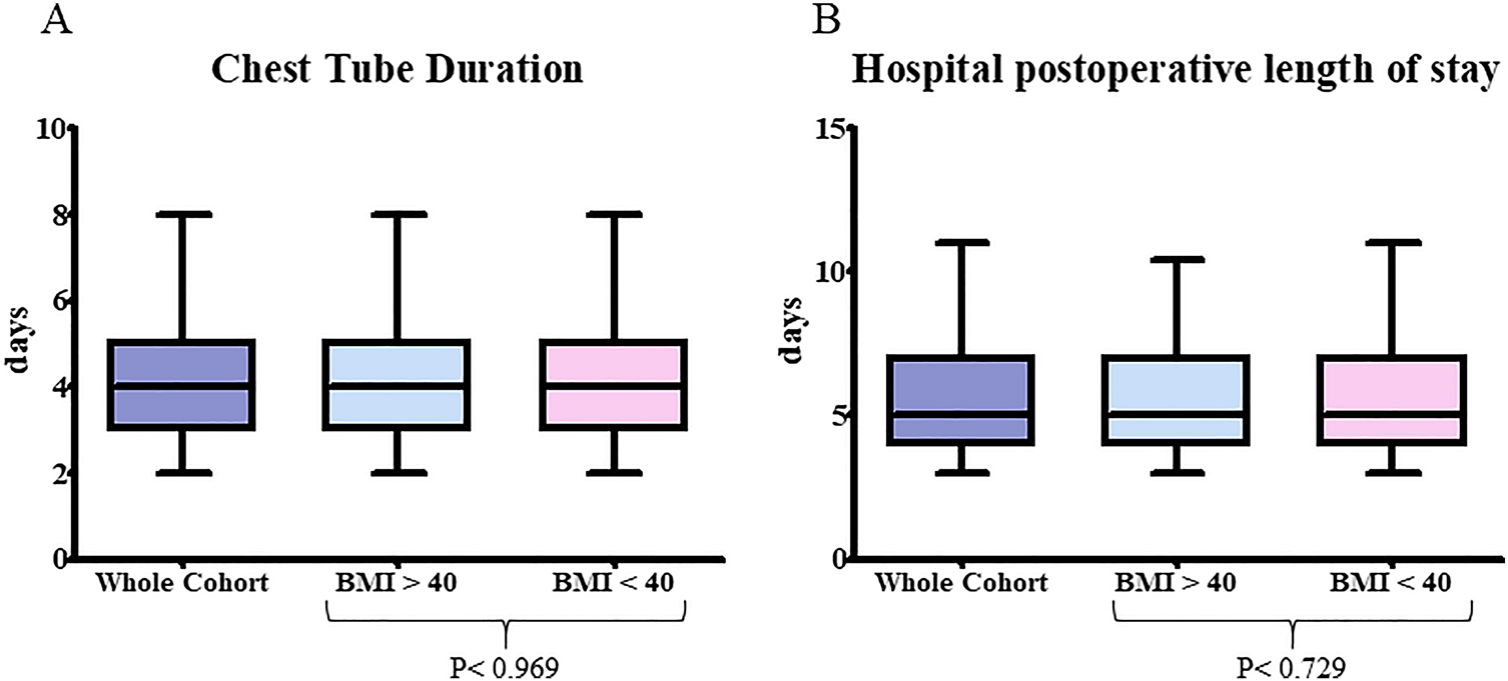
Morbid obesity (BMI ≥ 40) Versus Non-morbid obesity (BMI < 40): Box-and-whisker plots illustrate the distribution of chest tube duration (**A**), hospital postoperative length of stay (**B**)

At the univariable analysis, the Morbid obesity group showed a significant higher post-operative morbidity rate (26 *vs* 880—35.1% *vs* 20.3%) [Odds Ratio (OR) 2.13, 95% C.I. 1.31–3.45, *P* = 0.002). The most frequent postoperative complications in morbidly obese patients were pulmonary-related (9–35%): 3 persistent pleural effusion/empyema, 2 prolonged air leak (> 7 days), 2 prolonged mechanical ventilation, 1 pulmonary embolism, 1 atelectasis. Morbid obesity was not associated with a higher rate of conversion (10 *vs* 415—13.5% *vs* 9.6%) (OR 1.48, 95% C.I. 0.75–2.90, *P* = 0.26) and surgical margin positivity rates (1 *vs* 99—1.4% *vs* 2.4%) (OR 0.58, 95% C.I. 0.080–4.23, *P* = 0.59). Morbid obesity patients presented an equivalent surgical time (180 min vs 173 min, *P* = 0.116), lymph-node retrieval (9 vs 11, *P* = 0.835), intraoperative blood loss (100 ml vs 100 ml, *P* = 0.554), chest tube duration (4 days vs 4 days, *P* = 0.969), and hospital post-operative length of stay (5 days vs 5 days, *P* = 0.729) than non-morbid obese patients (Figs. 1 and 2).

At the multivariable analyses (Table 3), Morbid obesity resulted to be an independent prognostic factor for postoperative morbidity rate (OR 2.74, 95% C.I. 1.63–4.61, *P* < 0.001). Morbid obesity was not associated with a higher rate of conversion (OR 1.63, 95% C.I. 0.79, 3.39, *P* = 0.19).

**Table 3:** Logistic regression models from primary endpoint: Morbid obesity (BMI ≥ 40) vs Non-morbid obesity (BMI < 40) groups

Factor	BMI ≥ 40	BMI < 40	Univariable model	*P*	Multivariable-adjusted model^a^	*P*
	*n* = 74	*n* = 4338				
Post-operative complications (yes), *n* (%)	26 (35.1)	880 (20.3)	OR 2.13, 95% C.I. 1.31–3.45	0.002	OR 2.74, 95% C.I. 1.63–4.61	< 0.001
Conversion to thoracotomy (yes), *n* (%)	10 (13.5)	415 (9.6)	OR 1.48, 95% C.I. 0.75–2.90	0.26	OR 1.63, 95% C.I. 0.79, 3.39	0.19
Surgical margin positivity (yes), *n* (%)	1 (1.4)	99 (2.4)	OR 0.58, 95% C.I. 0.080–4.23	0.59	OR 0.67, 95% C.I. 0.089, 5.07	0.70

*BMI* Body Mass Index

^a^Adjusted for age, gender, smoking history, CCI, ECOG performance status, FEV1%, DLCO%, surgeon experience, pT stage, pN stage, preoperative diagnosis, and performed adhesiolysis

The additional multivariable analysis showed morbid obesity as independent prognostic factor for morbidity rate (BMI ≤ 30 as reference; BMI 30–40 OR 1.34, 95% C.I. 0.87, 2.07, *P* = 0.16; ≥ 40 OR 2.74, 95% C.I. 1.63–4.59, *P* < 0.001), but not for conversion rates (BMI ≤ 30 as reference; BMI 30–40 OR 1.48, 95% C.I. 0.86, 2.56, *P* = 0.16; ≥ 40 OR 1.59, 95% C.I. 0.77, 3.27, *P* = 0.21).

## Discussion

The minimally invasive surgical approaches have been established in order to increase perioperative outcomes and decrease postoperative complications, with respect to the standard open approaches.

Also, in thoracic surgery, these approaches [i.e., VATS and robotic-assisted thoracic surgery (RATS)] should be advisable especially in the high-risk surgical populations, like the elderly and obese ones [18]. Presumably, the ratio of patients with elevated BMI referred to thoracic surgical procedures will constantly increase in the next future [11, 14]. Consequently, it is mandatory to obtain a comprehensive insight into the effect of morbid obesity on perioperative outcomes in such patients [11, 14].

The results of the present study suggest that, in the cohort from the Italian VATS Group database,

1. Morbid obesity was not associated with a higher rate of conversion and surgical margin positivity rates.
2. Morbid obesity was associated with a higher rate of complications, in particular with the pulmonary-related one.
3. Morbid obesity patients benefit from an equivalent surgical time, lymph-node retrieval, intraoperative blood loss, hospital postoperative length of stay, and chest tube duration than non-morbid obese patients.

Normally, patients with increased BMI are characterized by the presence of cardiovascular comorbidities, which could jeopardize hemodynamic stability. Similarly, obese patients present a decrease of residual capacity, augmented airway resistance, and a reduction of chest wall compliance, which may increase the risk of pulmonary complications [4, 19–22]. Furthermore, more than 40% of obese patients had Obstructive Sleep Apnea Syndrome [23]. OSAS patients present the incapacity to maintain airway patency, with intermittent respiratory obstruction and intensification of respiratory efforts. Finally, drug metabolism could strongly differ between patients with normal and increased BMI; consequently, titration and careful dosing should be mandatory. For all these reasons, it could be intuitive that patients with elevated BMI developed more frequently post-operative complications. Nevertheless, the current literature presents contradictory results on this topic, and the “obesity paradox,” namely the protective effect of obesity on complication incidence, has been observed also in the thoracic surgery cohort analysis [24–28]. On the other hand, several studies presented a higher incidence of complications (notably pulmonary ones) in obese patients submitted to thoracic surgery [12, 21, 29].

The foundation of the obesity paradox has not been clearly elucidated, but protective effect peripheral body fat and reduced inflammatory response are common assumptions reported in the literature [10]. Nevertheless, Childers and Allison [30] proposed a mathematical model (U-shaped curve) in order to explain this occurrence: the highest mortality was presented by severe BMI values (both morbid obesity and severe underweight), while overweight, light, or moderate obesity shown lower mortality rate. Indeed, Tulinskýc et al. suppose that if the ratio of morbidly obese patients in their cohort was higher, the occurrence of complications would have been higher [28]. Coherently, in the present study, we focalize on morbidly obese patients. Our results showed that morbid obesity was associated with a higher rate of complications after VATS lobectomy, with a high rate of pulmonary-related complications. In particular, only 30% of the pulmonary complications observed in Morbid could be considered major one. Nevertheless, greater care and attention must be paid in the early recognition and treatment of this kind of morbidity, that this particularly related to obesity pathophysiology [5].

Interestingly, we did not find an association between morbid obesity and other perioperative clinical or technical outcomes, as the rate of conversion, surgical time, and surgical margin positivity rates, lymph-node retrieval, intraoperative blood loss, hospital postoperative length of stay, and chest tube duration. In particular, our findings are in contrast with St Julien et al. that investigated the database of the society of thoracic surgeons and observed an increased operating time by 7.2 min for every 10-unit increase in BMI [29]. On the other hand, our results were in line with a recent study on RATS lobectomy that demonstrated equivalence in surgical time between obese and non-obese patients [24].

The present study could be affected by several limitations, principally associated with a large multi-institutional dataset setting, and the retrospective nature of the research. Nevertheless, the major strength of our analysis is the use of a large, homogeneous, and prospectively maintained national scale patient cohort, as the one proved by the Italian VATS Group database. This fact permits the data reliability and, consequently, reinforces our conclusions.

To conclude, our findings showed that VATS lobectomy could be safely and satisfactorily conducted even in morbidly obese patients, without an increase in conversion rate, blood loss, surgical time, hospital postoperative length of stay, and chest tube duration. Moreover, short-term technical and oncological outcomes were preserved. Nevertheless, greater care and attention must be paid to the possible development of morbidity, and in particular pulmonary ones, in the postoperative period.

## References

[1] https://ec.europa.eu/eurostat/statistics-explained/index.php/Overweight_and_obesity_-_BMI_statistics. Accessed 1 Nov 2020

[2] World Health Organization. Obesity and Overweight (2020) http://www.who.int/news-room/fact-sheets/detail/obesity-and-overweight. Accessed 1 Nov 2020

[3] Lauro R, Sbraccia P, Lenzi A (2021) Italian Obesity Barometer Report. Obesity Monitor, vol 3. IBDO Foundation, Rome

[4] Chau EH, Lam D, Wong J, Mokhlesi B, Chung F (2012). Obesity hypoventilation syndrome: a review of epidemiology, pathophysiology, and perioperative considerations. Anesthesiology.

[5] Abumrad NA, Klein S (2010). Update on the pathophysiology of obesity. Curr Opin Clin Nutr Metab Care.

[6] Stevens J, Cai J, Pamuk ER (1998). The effect of age on the association between body-mass index and mortality. N Engl J Med.

[7] Haslam DW, James WP (2005). Obesity. Lancet.

[8] Flegal KM, Kit BK, Orpana H, Graubard BI (2013). Association of all-cause mortality with overweight and obesity using standard body mass index categories: a systematic review and meta-analysis. JAMA.

[9] Bamgbade OA, Rutter TW, Nafiu OO, Dorje P (2007). Postoperative complications in obese and nonobese patients. World J Surg.

[10] Valentijn TM, Galal W, Hoeks SE, van Gestel YR, Verhagen HJ, Stolker RJ (2013). Impact of obesity on postoperative and long-term outcomes in a general surgery population: a retrospective cohort study. World J Surg.

[11] Liou DZ, Berry MF (2018). Thoracic surgery considerations in obese patients. Thorac Surg Clin.

[12] Attaran S, McShane J, Whittle I, Poullis M, Shackcloth M (2012). A propensity-matched comparison of survival after lung resection in patients with a high versus low body mass index. Eur J Cardiothorac Surg.

[13] Jagoe RT, Goodship TH, Gibson GJ (2001). Nutritional status of patients undergoing lung cancer operations. Ann Thorac Surg.

[14] Ferguson MK, Vigneswaran WT (2008). Changes in patient presentation and outcomes for major lung resection over three decades. Eur J Cardiothorac Surg.

[15] Lim E, Batchelor T, Shackcloth M, Dunning J, McGonigle N, Brush T, Dabner L, Harris R, Mckeon HE, Paramasivan S, Elliott D, Stokes EA, Wordsworth S, Blazeby J, Rogers CA, VIOLET Trialists (2019). Study protocol for VIdeo assisted thoracoscopic lobectomy versus conventional open LobEcTomy for lung cancer, a UK multicentre randomised controlled trial with an internal pilot (the VIOLET study). BMJ Open.

[16] Solli P, Bertolaccini L, Droghetti A, Bertani A, Gonfiotti A, Nosotti M, Migliore M, Crisci R, VATS Group I (2018). 2016 annual report from the Italian VATS group. Future Oncol.

[17] Dindo D, Demartines N, Clavien PA (2004). Classification of surgical complications: a new proposal with evaluation in a cohort of 6336 patients and results of a survey. Ann Surg.

[18] Guerrera F, Olland A, Ruffini E, Falcoz PE (2019). VATS lobectomy vs. open lobectomy for early-stage lung cancer: an endless question-are we close to a definite answer?. J Thorac Dis..

[19] Poirier P, Alpert MA, Fleisher LA (2009). Cardiovascular evaluation and management of severely obese patients undergoing surgery: a science advisory from the American heart association. Circulation.

[20] Pedoto A (2012). Lung physiology and obesity: anesthetic implications for thoracic procedures. Anesthesiol Res Pract.

[21] Launer H, Nguyen DV, Cooke DT (2013). National perioperative outcomes of pulmonary lobectomy for cancer in the obese patient: a propensity score matched analysis. J Thorac Cardiovasc Surg.

[22] Gurevich-Panigrahi T, Panigrahi S, Wiechec E, Los M (2009). Obesity: pathophysiology and clinical management. Curr Med Chem.

[23] Busetto L, Enzi G, Inelmen EM (2005). Obstructive sleep apnea syndrome in morbid obesity: effects of intragastric balloon. Chest.

[24] Montané B, Toosi K, Velez-Cubian FO, Echavarria MF, Thau MR, Patel RA, Rodriguez K, Moodie CC, Garrett JR, Fontaine JP, Toloza EM (2017). Effect of obesity on perioperative outcomes after robotic-assisted pulmonary lobectomy. Surg Innov.

[25] Thomas PA, Berbis J, Falcoz PE, Le Pimpec-Barthes F, Bernard A, Jougon J, Porte H, Alifano M, Dahan M, EPITHOR Group (2014). National perioperative outcomes of pulmonary lobectomy for cancer: the influence of nutritional status. Eur J Cardiothorac Surg..

[26] Wang C, Guo M, Zhang N, Wang G (2018). Association of body mass index and outcomes following lobectomy for non-small-cell lung cancer. World J Surg Oncol.

[27] Paul S, Andrews W, Osakwe NC, Port JL, Lee PC, Stiles BM, Altorki NK (2015). Perioperative outcomes after lung resection in obese patients. Thorac Cardiovasc Surg.

[28] Tulinský L, Mitták M, Tomášková H, Ostruszka P, Penka I, Ihnát P (2018). Obesity paradox in patients undergoing lung lobectomy—myth or reality?. BMC Surg.

[29] St Julien JB, Aldrich MC, Sheng S (2012). Obesity increases operating room time for lobectomy in the society of thoracic surgeons database. Ann Thorac Surg.

[30] Childers DK, Allison DB (2010). The ‘obesity paradox’: a parsimonious explanation for relations among obesity, mortality rate and aging?. Int J Obes.

